# Transcript profiling of common bean (*Phaseolus vulgaris *L.) using the GeneChip^® ^Soybean Genome Array: optimizing analysis by masking biased probes

**DOI:** 10.1186/1471-2229-10-85

**Published:** 2010-05-07

**Authors:** S Samuel Yang, Oswaldo Valdés-López, Wayne W Xu, Bruna Bucciarelli, John W Gronwald, Georgina Hernández, Carroll P Vance

**Affiliations:** 1 USDA-Agricultural Research Service, Plant Science Research, St Paul, MN 55108, USA; 2 Centro de Ciencias Genómicas, Universidad Nacional Autónoma de México, Ap. Postal 565-A, 62210 Cuernavaca, Mor. México; 3 Supercomputing Institute for Advanced Computational Research, University of Minnesota, Minneapolis, MN 55455, USA; 4 Department of Agronomy and Plant Genetics, University of Minnesota, St. Paul, MN 55108, USA

## Abstract

**Background:**

Common bean (*Phaseolus vulgaris *L.) and soybean (*Glycine max*) both belong to the *Phaseoleae *tribe and share significant coding sequence homology. This suggests that the GeneChip^® ^Soybean Genome Array (soybean GeneChip) may be used for gene expression studies using common bean.

**Results:**

To evaluate the utility of the soybean GeneChip for transcript profiling of common bean, we hybridized cRNAs purified from nodule, leaf, and root of common bean and soybean in triplicate to the soybean GeneChip. Initial data analysis showed a decreased sensitivity and accuracy of measuring differential gene expression in common bean cross-species hybridization (CSH) GeneChip data compared to that of soybean. We employed a method that masked putative probes targeting inter-species variable (ISV) regions between common bean and soybean. A masking signal intensity threshold was selected that optimized both sensitivity and accuracy of measuring differential gene expression. After masking for ISV regions, the number of differentially-expressed genes identified in common bean was increased by 2.8-fold reflecting increased sensitivity. Quantitative RT-PCR (qRT-PCR) analysis of 20 randomly selected genes and purine-ureide pathway genes demonstrated an increased accuracy of measuring differential gene expression after masking for ISV regions. We also evaluated masked probe frequency per probe set to gain insight into the sequence divergence pattern between common bean and soybean. The sequence divergence pattern analysis suggested that the genes for basic cellular functions and metabolism were highly conserved between soybean and common bean. Additionally, our results show that some classes of genes, particularly those associated with environmental adaptation, are highly divergent.

**Conclusions:**

The soybean GeneChip is a suitable cross-species platform for transcript profiling in common bean when used in combination with the masking protocol described. In addition to transcript profiling, CSH of the GeneChip in combination with masking probes in the ISV regions can be used for comparative ecological and/or evolutionary genomics studies.

## Background

Common bean (*Phaseolus vulgaris *L.), an herbaceous annual legume, is one of the most ancient crops of the New World. Like other legumes, common bean can acquire nitrogen through mutualistic symbiosis with nitrogen (N)-fixing bacteria of the family *Rhizobiaceae*[1,2]. Common bean is the most important economic variety of the genus *Phaseolus *and is grown widely in all parts of the world. According to a recent report, over 17.99 million metric tons were produced in the world in 2008 [3]. It is the most important grain legume for direct human consumption comprising about 50% of the grain legumes consumed world-wide [4]. It is also the primary source of dietary protein in many developing countries[5].

A high-density oligonucleotide microarray is not yet available for global transcript profiling of common bean. Previously, a macro array that contained a total of 1,786-unigene sets derived from a common bean nodule cDNA library was used for transcript profiling of different organs such as root, leaf, stem, and pod [6]. Currently available is the GeneChip^® ^Soybean Genome Array (soybean GeneChip). The soybean GeneChip contains over 37,500 probe sets designed from soybean transcripts. Additionally, the soybean GeneChip contains 15,800 and 7,500 probe sets designed from *Phytophthora sojae *(a water mold that commonly attacks soybean crops) and *Heterodera glycines *(a *cyst nematode *pathogen) transcripts, respectively. Common bean (2n = 2x = 22) is a diploid relative of soybean (2n = 2x = 40) and soybean is considered to be a diploidized tetraploid species (ancient polyploid). Recent release of the soybean genome sequence revealed a highly duplicated soybean genome where about 75% of the genes are represented in multiple copies [7]. Contrary to the highly duplicated soybean genome, most of the genetic markers in common bean were present as a single copy [8-10]. However, previous studies reported a significant level of colinearity in gene order among the tropical legumes such as common bean and soybean even though both genomes might have undergone extensive gene diversification and loss, and numerous chromosome rearrangements [7,11-13]. A previous study also suggested common bean as a reference model to study the dynamics of genome evolution and as an additional resource for gene discovery in soybean [14]. The close relationship between soybean and common bean suggests that the soybean GeneChip may be used for transcript profiling in common bean.

Microarrays developed for a specific species have been used for transcript profiling of closely related species. Cross-species RNA hybridization (CSH) to DNA microarrays has been used successfully in both animal and plant when a representative microarray platform is not available [15-34]. The assumption that underlies the validity of CSH on a gene chip of a closely related species is that the level of sequence homology among genes conserved between closely related species is significant enough to enable the detection of messages by probes originally designed for their orthologs. However, caution must be taken when interpreting the CSH results. Notably, factors such as sequence divergence, alternative splicing, and cross-hybridization can cause spurious variation in signal intensity leading to bias in transcript profiling. Such aberrant variation derived from CSH result in decreased sensitivity and accuracy of measuring differential gene expression that is typically reflected by a decrease in number of genes detected and a compressed fold change difference, respectively. It is therefore necessary to find and keep truly informative probes and eliminate biased probes prior to the microarray data analysis.

Microarrays spotted with cDNAs or longer oligos can be used for CSH studies as well. However, the GeneChip with 25-mer oligos has an advantage over microarrays with cDNAs or longer oligos. In general, shorter oligos are more sensitive to sequence mismatch but longer oligos (or cDNAs) tend to endure sequence mismatch during hybridization. In addition, each probe set in the GeneChip consists of 11 perfect match (PM) probes (25-mer) that target mostly the non-overlapping portion of the coding region for the corresponding target gene (probe set). The expression values of target genes (probe sets) are derived from the signal intensities of those 11 PM probes using the signal condensing methodology such as the Microarray Suite (MAS) 5.0 (http://www.affymetrix.com) or the Robust Multi-array Average algorithm (RMA) [35]. Since the minimum number of probes required to produce (or retain) the expression value of a probe set is one, the putative biased probes (i.e., probes targeting ISV region) can be electronically masked (or flagged) and only truly informative probes with good signal intensity can be used for producing the expression values of the probe sets. However, this is impossible for arrays with cDNA or longer oligos where elimination of a cDNA (or long oligos) will result in the loss of the corresponding target gene on the array.

There have been numerous efforts to optimize CSH GeneChip data. For example, Ranz et al. [20] introduced a genomic DNA hybridization-based method that selected conserved probes between target and non-target species. Only the conserved probes selected were used for transcript profiling in non-target species. Several CSH studies have utilized this approach [20,29,32]. However, a recent study questioned the reliability of the DNA hybridization-based method for selecting unbiased probes in CSH studies [34]. Wang et al. [27] took a different approach to identify inter-species conserved (ISC) probe sets based on the expressed sequence tag (EST) homology between target and non-target species. However, this approach is not suitable for species with limited EST resources such as common bean. Ji et al. [23] developed a different optimization technique based on masking probes with poor signal intensities in a CSH GeneChip data.

In this study, we used the soybean GeneChip for transcript profiling in three different organs (nodule, root, and leaf) of both common bean and soybean. We optimized CSH GeneChip data analysis by masking putative probes targeting interspecies variable regions. Transcript profiling and qRT-PCR data suggests that the soybean GeneChip is a suitable cross-species platform for transcript profiling in common bean when used in combination with the masking protocol described.

## Results and Discussion

To determine the sequence similarities between soybean and common bean and to predict the efficacy of the GeneChip^® ^Soybean Genome Array (Affymetrix Inc., Santa Clara, CA) for transcript profiling of common bean, the soybean probe set target sequences (http://www.affymetrix.com) were blasted against 21,497 unique sequences in the common bean gene index [36]. A total of 18,401 putative orthologous bean gene sequences were identified (an e-value cutoff of 1e-10). The orthologs from the two species were on average ~89% identical at the DNA sequence level (data not shown). However, when 671,762 PM soybean probe sequences (25-mer, each probe set in the soybean GeneChip contains 11 PM probes) on the soybean GeneChip (http://www.affymetrix.com) were blasted against the common bean gene index sequences with an e-value cutoff of 0.1, a total of 134,876 probe sequences had hits among bean sequences. About 38% of them showed at least one base mismatch over the 16-25 bp alignment (data not shown). These probes targeting ISV regions can cause problems when using the soybean GeneChip for transcript profiling of common bean. In general, the effect of using a cross-species platform for global transcript profiling of a closely-related species is 1) decreased sensitivity (number of genes detected) and 2) decreased accuracy of measuring differential gene expression (compressed fold-change).

### Overview of the GeneChip data

To evaluate the utility of the GeneChip^® ^Soybean Genome Array (soybean GeneChip) for transcript profiling of common bean, we hybridized cRNA purified from nodule, leaf, and root of common bean and soybean, in triplicate, to the soybean GeneChip (18 GeneChip hybridizations = 2 species × 3 organs × 3 replicates). To provide an overview of probe set detection call rate ('present' vs. 'absent') for each organ in each species, we condensed the probe signal intensities for each probe set in each GeneChip hybridization using the MAS 5.0 http://(http://www.affymetrix.com). On average, MAS 5.0 called 42.8%, 45.1%, and 49.1% of the probe sets on the GeneChip as "present" for soybean nodule, leaf, and root, respectively. However, the present call rates for the same organs in common bean were decreased to 15.2%, 15.7%, and 17.7%, respectively. The significantly reduced present call rates for common bean reflects a decreased sensitivity to predict a valid transcript profile of common bean.

**Figure 1:**
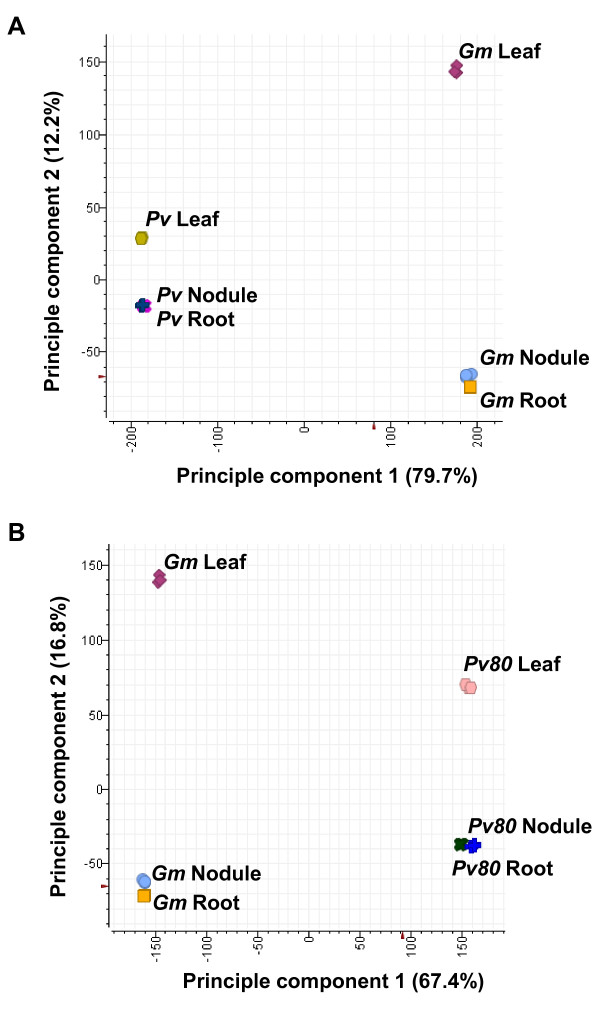
**Principal component analysis of the GeneChip data from nodule, leaf, and root tissue samples of common bean and soybean before (A) and after (B) masking probes with a signal intensity threshold of 80**. The first and second principal components together accounted for about 92% and 84% of the total variation in the data before and after masking, respectively. The percentages represent the variation explained by each principal component. Gm, *Glycine max*; Pv, *Phaseolus vulgaris *L.; Pv80, *Phaseolus vulgaris *L. after masking with signal intensity threshold 80.

To explore the overall structure of the GeneChip data in terms of the variance components, we performed a principal component analysis (PCA) on the common bean and soybean GeneChip data. The first principal component accounted for 79.7% of the total variability in the data with the two species separated along the first principal component axis (Figure 1A). The second principal component accounted for 12.2% of the total variability in the data with leaf tissue separated from nodule and root tissue along the second principal component axis (Figure 1A). The results from the PCA suggested that the variation between the two species is more significant than the variation among different organs. It also suggested that the leaf tissue had a more distinct gene expression pattern compared to nodule and root tissue. Nodule and root tissue shared similar gene expression patterns. The large variation between the two species (79.7% of the total variability) may have been caused by the significantly decreased hybridization signal intensity for common bean compared to that for soybean. The shorter distance between leaf and nodule (or root) along the second principal component axis in common bean compared to that of soybean suggested a decreased accuracy of measuring differential gene expression in common bean CSH GeneChip data compared to that of soybean.

### Masking probes targeting inter-species variable (ISV) regions

**Figure 2:**
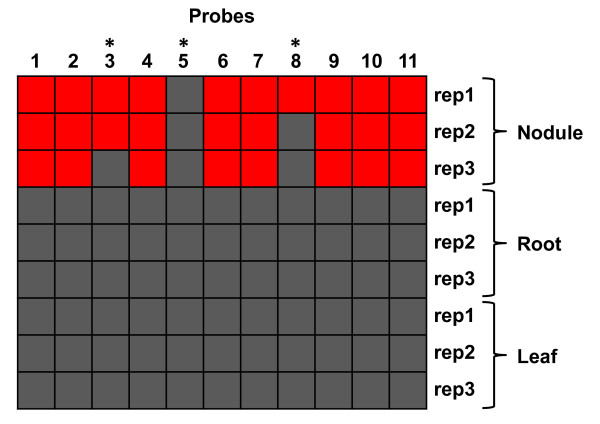
**Masking putative probes targeting ISV regions**. For each common bean tissue sample, three biological replicates were collected producing a total of 9 data points per probe (3 tissue types × 3 replicates). For a particular probe, all data points were kept if three or more signals were above the signal intensity threshold. Otherwise, all 9 signals were masked (see Methods for details). Red and grey squares represent signals above and below the masking signal intensity threshold, respectively. Asterisk, probes with less than 3 signals above the masking signal intensity threshold.

**Figure 3:**
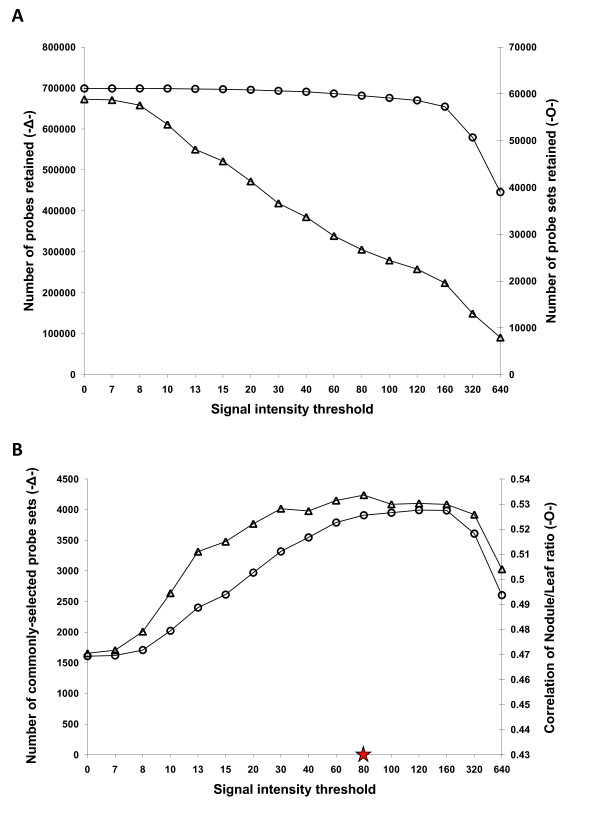
**Selection of the optimum signal intensity threshold for masking probes targeting interspecies-variable regions**. A. The number of probes (triangles) and probe sets (circles) retained after masking probes with a series of signal intensity thresholds. B. Effect of probe masking over a range of signal intensity thresholds (0-640) on the number of probe sets commonly-selected in soybean and common bean (circles) and the correlation of the Leaf/Nodule hybridization intensity ratio for the commonly selected genes (triangles). A signal intensity threshold of 80 (red star) was selected to mask biased probes. Commonly-selected genes are defined as genes exhibiting at least a 2-fold difference in hybridization intensity expression ratio between leaf and nodule tissue (leaf vs. nodule, ≥ 2-fold difference) for both soybean and common bean.

As an initial step to optimize the common bean CSH GeneChip data, we used a series of hybridization signal intensity thresholds (5, 7, 8, 10, 13, 15, 20, 30, 40, 60, 80, 100, 120, 160, 320, 640) to mask probes with signals below the threshold. For each common bean tissue sample, three biological replicates were collected producing a total of 9 data points per probe (3 tissue types × 3 replicates). For a particular probe (9 data points), all data points were kept if three or more signals were above the signal intensity threshold. Otherwise, all 9 signals were masked (see Methods for details). Next, the signal intensities of retained probes were quantile-normalized and condensed into probe set expression values by RMA [35]. Figure 2 shows the hybridization patterns for a hypothetical probe set (3 tissue types × 3 replicates). Among the 11 PM probes in a probe set, 8 probes with more than 3 signals (out of 9 data points) above the masking signal intensity threshold (red squares) were kept for producing the probe set expression value for each replicate in each tissue type by RMA. However, 3 probes (*) with less than 3 signals above the masking signal intensity threshold were masked and not included for RMA (Figure 2). As expected, the number of probes retained decreased rapidly (from ~672,870 to ~90,789) while the number of probe sets retained decreased gradually (from ~61,170 to ~39,000) as signal intensity threshold increased from zero to 640 (Figure 3A).

In a previous study, Ji et al. [23] developed a masking protocol to selectively mask probes with poor signal intensity in a probe set putatively targeting ISV regions of the probe set target sequence in the CSH GeneChip data. To evaluate whether masking improved accuracy of measuring differential gene expression, it was hypothesized that different organs (heart, liver) of humans and non-human mammals have similar gene expression patterns. After masking low intensity probes in the CSH microarray data, Ji et al. [23] found an improved correlation for Ln(heart/liver) values between human and mouse GeneChip data. These authors concluded that comparisons of gene expression patterns in defined tissues of related species could be used to optimize CSH studies involving other mammals or plants. In another previous study, Yang et al. [37] conducted transcript profiling using the *Medicago *GeneChip^® ^with elongating stem (ES) and post-elongation stem (PES) internodes from alfalfa genotypes 252 and 1283 that differ in stem cell wall concentrations of cellulose and lignin. To optimize the alfalfa CSH GeneChip data, Yang et al. [37] developed a protocol to mask probes with poor signal intensity in a probe set putatively targeting ISV regions of the probe set target sequence. The masking protocol developed was based on the assumption that the ratio of gene expression in ES and PES internodes (PES/ES) of alfalfa is very similar to that measured in tissues of *Medicago truncatula*. Yang et al. [37] selected a masking signal intensity threshold that maximized the number of differentially expressed genes (PES/ES ≥ 2-fold difference) commonly selected in both species while maintaining a high correlation coefficient of the PES/ES ratio of commonly selected genes between two species. By employing the masking protocol before the CSH GeneChip data analysis, the problems associated with transcript profiling in alfalfa stems using the Medicago GeneChip as a CSH platform were mitigated [37].

The masking protocol employed in this study is based on the assumption that the overall gene expression pattern for common bean nodule and leaf tissue is very similar to that measured in the same tissue of soybean [23,37]. In this study, soybean GeneChip data for nodule and leaf tissue was analyzed in parallel with the common bean CSH GeneChip data masked by a series of signal intensity thresholds to select a masking signal intensity threshold that could optimize both sensitivity and accuracy of measuring differential gene expression. To evaluate the effect of masking on sensitivity, we identified genes (probe sets) with at least a 2-fold difference in expression between soybean leaf and nodule. Next, we identified genes with at least a 2-fold difference in expression between common bean leaf and nodule after masking with each signal intensity threshold. Differentially expressed genes (nodule/leaf ≥ 2-fold difference) commonly identified in both soybean and common bean were referred to as "commonly-selected genes" (Figure 3B). The number of commonly-selected genes increased (from ~1,608 to ~3,992) as the signal intensity threshold increased to a value of 120. This reflected an increase in detection sensitivity (Figure 3B). To evaluate the effect of masking on accuracy of measuring differential gene expression, we examined the correlation of the nodule/leaf signal ratio for the commonly-selected genes between two species as the signal intensity threshold was increased. The Pearson correlation coefficient of the nodule/leaf ratio between soybean and common bean increased from 0.47 to 0.53 as the signal intensity threshold increased to a value of 80. This reflected an increase in accuracy of measuring differential gene expression (Figure 3B). The number of commonly-selected genes and the calculated Pearson correlation coefficient significantly declined at signal intensity thresholds above 160.

Although the highest number of commonly-selected genes was achieved with a signal intensity threshold of 120, the Pearson correlation coefficient of the nodule/leaf ratio between soybean and common bean was reduced (Figure 3B). However, with a signal intensity threshold of 80 the highest correlation was achieved, with over 59,600 probe sets (about 97% of the total number on the soybean GeneChip) retained (Figure 3A). In addition, over 96% of the soybean probe sets were retained with a signal intensity threshold 80 (data not shown). On the basis of these results, we decided to use a signal intensity threshold of 80 for masking biased probes targeting ISV regions. The use of this masking threshold significantly improved sensitivity (the number of expressed genes detected) while maintaining a high level of accuracy of measuring differential gene expression (accuracy of fold-difference detection).

**Figure 4:**
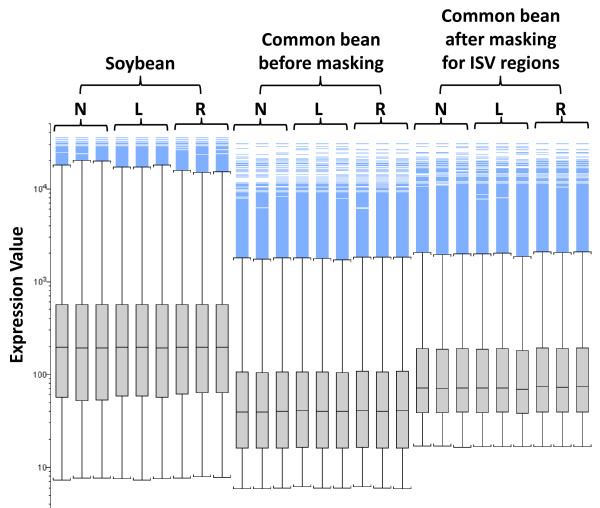
**Box plots of 9 GeneChip data sets (3 tissue types × 3 replications) from soybean and common bean before and after masking for ISV regions (signal intensity threshold = 80)**. Blue lines simply represent outliers.

The effect of masking biased probes of the common bean CSH GeneChip data with a signal intensity threshold of 80 is shown in Figure 4. Overall, masking ISV regions increased the signal intensity of the common bean probe sets (~2-fold). Figure 1B shows the effect of masking biased probes with a signal intensity threshold of 80 on the overall structure of the GeneChip data. The PCA data that was generated after masking showed a similar pattern as the one generated before masking, i.e. soybean and common bean GeneChip data sets were separated along the first principal component axis and the second principal component axis separated leaf from nodule and root (Figure 1B).  Interestingly, the first principal component generated after masking accounted for 67.4% of the total variability in the GeneChip data (Figure 1B). This is a significant reduction from the 79.7% prior to masking (Figure 1A). Additionally, after masking the second principal component accounted for 16.8% of the total variability in the GeneChip data, a significant increase from 12.2% prior to masking (Figure 1A and 1B). The reduced overall variability between the two species and increased variability among different tissues of these species suggested increased accuracy of measuring differential gene expression in common bean CSH GeneChip data after masking with the signal intensity threshold of 80.

To test the effectiveness of the masking strategy employed in this study for flagging probes targeting the ISV regions, we examined the homology between soybean probe targets (25-mer) and common bean EST sequences using the BLASTN program. A signal intensity threshold of 80 was also used for this examination. A total of 85,118 out of 301,621 soybean probes retained with a signal intensity threshold of 80 had common bean EST hits. A total of 48,885 out of 367,256 soybean probes masked with the signal intensity threshold of 80 also had common bean EST hits. Interestingly, the average number of mismatched bases between the soybean probe target sequences and common bean ESTs for the masked probes was over twice the number of the retained probes (0.45 vs. 1). This result verifies the effectiveness of the masking strategy applied in this study for flagging putative biased probes targeting ISV regions. Although these results provide support for the validity of the masking methodology used in this study, caution needs to be exercised in interpreting the data. The comparison is based on the limited number of common bean EST sequences that are currently available in the public database. Another factor to consider in the interpreting these results is the observation that location of a sequence mismatch can influence the degree of probe hybridization. Only sequence mismatches located near the center of a probe can affect target hybridization and probes often can endure mismatches located near the ends. Thus, the number of mismatches per probe cannot absolutely reflect probe target hybridization efficiency in a CSH study. In addition, the masking protocol used in this study is only an optimization tool, not an absolute solution for correcting bias in the CSH GeneChip data. For example, intensity-based masking protocol can be biased toward abundant transcripts. It could over-mask probes as well even though the masking signal intensity threshold determined the maximized number of commonly-selected genes in both species while maintaining a high correlation coefficient for the Nodule/Leaf ratio of commonly-selected genes between the two species. Thus, for the candidate genes selected for downstream experiments, such as functional characterization, it is important to validate the CSH GeneChip data using other techniques such as qRT-PCR.

### Effect of masking on detection of differentially-expressed genes

**Figure 5:**
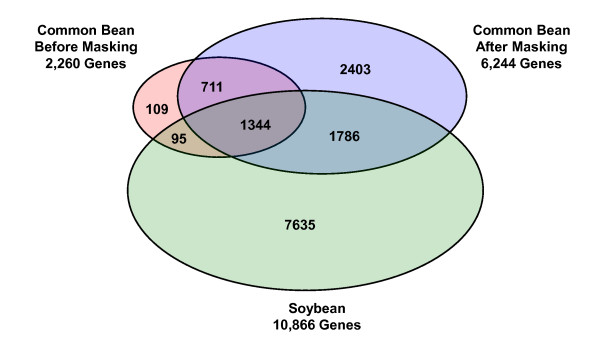
**Venn diagram showing numbers of overlapping and non-overlapping genes differentially expressed among three different organs of soybean and common bean before masking and after masking for ISV regions (threshold = 80)**. Differentially expressed genes were identified after an ANOVA (*p*-value < 0.0001, FDR < 0.0015) with an additional cutoff of a 2-fold ratio in pair-wise comparisons (i.e., nodule vs. leaf; nodule vs. root; root vs. leaf).

We used an ANOVA (*p*-value < 0.0001, FDR < 0.0015) with an additional cutoff of a 2-fold ratio in a pair-wise comparison (i.e., nodule vs. leaf; nodule vs. root; root vs. leaf) to identify genes that were differentially expressed within taxa among three different organs of soybean (Additional file 1) and common bean (Additional file 2). The Venn diagram in Figure 5 shows the number of differentially expressed genes identified. Masking biased probes using a signal intensity threshold of 80 increased the number of differentially-expressed genes identified in common bean by 2.8-fold (2,260 vs. 6,244). Over 90% of the genes originally identified before masking were also identified after masking with an additional 4,189 newly identified genes (Figure 5). We estimate that the 204 genes identified prior to masking, but not included after masking, might have been putative false positives; 4,189 genes newly detected after masking might have been putative false negatives caused by ISV regions in the original GeneChip data (Figure 5).

### Validation of masking protocol by qRT-PCR with 20 randomly selected genes

**Figure 6:**
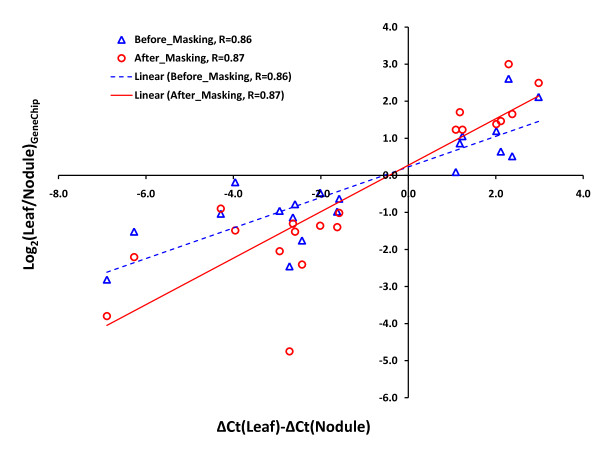
**qRT-PCR validation of the common bean CSH GeneChip data**. A total of 20 randomly selected genes were used for qRT-PCR validation. ΔΔCT values obtained from the qRT-PCR data were plotted against log_2_(Leaf/Nodule) hybridization intensity ratio values from the GeneChip data before (blue triangles) and after (red circles) masking. R, Pearson correlation coefficient.

To independently validate the masking protocol employed and the GeneChip data produced in this study, we performed qRT-PCR of 20 genes randomly selected from a list of genes identified before and after masking (Figure 5). Among the 20 genes selected for validation, 5 were detected only before masking, 5 were detected both before and after masking, and 10 were detected only after masking. We plotted ΔΔC_T _values obtained from the qRT-PCR data (*x-axis*) against log_2_(Leaf/Nodule) ratio values from the GeneChip data from both before (Δ) and after (o) masking (*y-axis*) (Figure 6). A previous study showed that the ΔΔC_T _value from qRT-PCR is linear to the log gene expression ratio between two samples [38]. The results showed a positive linear relationship between the ΔΔC_T _value from qRT-PCR and the log ratio both before and after masking (Figure 6). Masking slightly increased the *Pearson *correlation coefficient (*R*) from 0.86 to 0.87 (Figure 6).

### Overview of the transcript profiles in common bean and soybean

Probe sets on the soybean GeneChip were functionally classified using the MapMan gene functional classification system [39] (see Methods for details). We performed a Fisher's exact test to identify functional classes over- or under-represented among differentially expressed genes in each species. With a given number of genes in each class, the Fisher's exact test examines whether the number of genes in each class occurred only by chance (see Methods for details). We identified numerous functional class over- or under-represented among genes up- or down-regulated in nodule compared to leaf and root tissue in common bean and soybean (Additional file 3). Gene functional class over-representation analysis revealed differences in transcript enrichment patterns between common bean and soybean. For example, fermentation and bZIP transcription factor family classes were over-represented among genes up-regulated in soybean but not in common bean nodules. On the other hand, brassinosteroid biosynthesis genes (BR6OXs) were significantly overrepresented among genes up-regulated in common bean but not in soybean nodules. A previous study involving common bean EST analysis also suggested a unique gene expression pattern in common bean nodules compared to nodules of other legume species such as *Medicago truncatula *and soybean [6]. Contrary to differences in transcript functional class representation in nodules of soybean and common bean, we also found many functional classes that were over-represented in both. For example, the purine biosynthesis class was significantly over-represented among genes up-regulated in both soybean and common bean nodules (Additional file 3).

### Purine pathway genes: marker genes up-regulated in both common bean and soybean nodules

**Figure 7:**
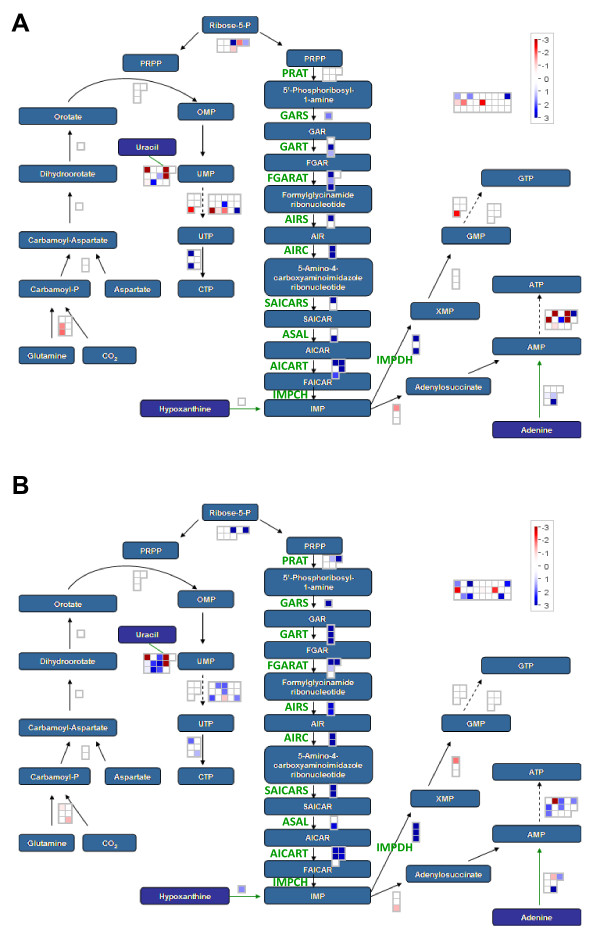
**MapMan overview of nucleotide synthesis showing the purine pathway genes that are preferentially expressed in nodule compared to leaf tissue in common bean (A) and soybean (B)**. Individual genes are represented by small squares. The Log2(nodule/leaf) values for the differentially expressed genes (p < 0.0001, FDR < 0.0015, ≥ 2-fold difference) were false color coded using a scale of -3 to ± 3. The intensity of blue and red colors indicates the degree of preferential expression of the corresponding genes in nodule and leaf, respectively. Color saturates at ± 3 (8-fold difference or higher). See Methods for details. A complete list of the differentially expressed genes, corresponding MapMan functional categories, signal intensities and log ratios are provided in additional file 1 and 2. Abbreviations: PRPP: phosphoribosyl pyrophosphate, PRAT: PRPP amidotransferase, GARS: GAR synthetase, GAR: glycinamide ribonucleotide, GART: GAR transformylase, FGAR: formylglycinamide ribonucleotide, FGARAT: FGAR amidotransferase, AIRS: AIR synthetase, AIR: aminoimidazole ribonucleotide, AIRC: AIR carboxylase, SAICARS: SAICAR synthetase, SAICAR: succinoaminoimidazolecarboximide ribonucleotide, ASAL: adenylosuccinate-AMP lyase, AICAR: aminoimidazolecarboximide ribonucleotide, AICART: AICAR transformylase, FAICAR: formylaminoimidazolecarboximide ribonucleotide, IMPCH: IMP cyclohydrolase, IMP: inosine monophosphate, IMPDH: IMP dehydrogenase.

In *rhizobi*a-infected nodules of tropical legumes such as soybean and common bean, nitrogen (N) fixed by bacterial nitrogenase in the form of NH_3 _or NH_4_^+ ^is assimilated by host plants initially through the amide (Gln) pathway. Subsequent steps involve the purine pathway which yields inosine monophosphate (IMP) followed by the formations of ureides (allantoin and allantoic acid). In these legumes, ureides synthesized in the nodules are loaded into the xylem, transported to the leaves, and used as a major source of N in the leaves. Thus, up-regulation of genes for the purine pathway enzymes in nodules compared to other organs is the most significant feature of the symbiosis in ureide-forming legumes [40,41]. The purine pathway involves 10 enzymatic steps with nine genes to synthesize IMP from phosphoribosyl pyrophosphate (PRPP) (Figure 7) [42]. These purine pathway genes are excellent marker genes for validating transcript profiling data involving nodule-specific or nodule-preferential gene expression. A MapMan overview of the purine biosynthesis pathway shows genes preferentially expressed in nodule (blue) versus leaf (red) in common bean (Figure 7A) and soybean (Figure 7B). In general, identical genes for enzymes in each step of the purine pathway were up-regulated in both common bean and soybean nodules. However, even after masking, decreased sensitivity and accuracy of measuring differential gene expression could still be observed in the common bean data. The results also show that common bean and soybean used different gene family members to catalyze steps in the purine pathway. For example, Gma.8500.1.S1_at, formylglycinamide ribonucleotide amidotransferase (FGARAT), and GmaAffx.33029.1.S1_at, aminoimidazolecarboximide ribonucleotide transformylase (AICART), were up-regulated in common bean but not in soybean nodules (Figure 7). To further test the validity of our CSH GeneChip data, we measured the expression of 13 purine-ureide pathway genes using qRT-PCR (Table 1). Among the 13 purine-ureide pathway genes selected for qRT-PCR validation, 8 were detected both before and after masking and 5 were detected only after masking. The qRT-PCR results revealed that all of the purine-ureide pathway genes examined in common bean were significantly up-regulated in nodule relative to leaf tissue. This suggests a significantly increased detection power (sensitivity) after masking (8 vs. 13 true positives) (Table 1). In addition, masking increased the *Pearson *correlation coefficient (*R*) between the ΔΔC_T _value from the qRT-PCR and the log ratio value from the CSH GeneChip data from -0.1 to 0.5 (Table 1). These *Pearson *correlation coefficient values produced before and after masking are significantly lower than those shown in Figure 6. This is because all of the purine-ureide pathway genes examined were up-regulated in nodules compared to leaf tissue within a narrow range of the expression ratio (0.1 - 3.8 before masking) (Table 1). Overall, the increased positive correlation between the two data sets after masking indicates the increase in accuracy of measuring differential gene expression obtained using the masking protocol.

**Table 1:** Purine-ureide pathway genes used for qRT-PCR validation.

		ANOVA^1^		**Log_2_(N/L)**^2^		
			
Annotation	Probe Set ID	No mask	Mask	No mask	Mask	ΔΔC_T_

GAR Synthetase [GARS (*pur2*)]	Gma.8431.1.S1_at	**-**	**+**	0.9	1.8	5.5
GAR transformylase [GART (*pur3*)]	Gma.16.1.S1_s_at	**+**	**+**	2.6	3.8	4.5
FGAR amidotransferase [FGARAT (*pur4*)]	Gma.12152.1.S1_at	**+**	**+**	3.8	4.1	5.3
AIR synthase [AIRS (*pur5*)]	Gma.5959.1.S1_at	**-**	**+**	1.3	4.7	4.1
AIR carboxylase [AIRC (*pur6*)]	Gma.7887.1.S1_at	**+**	**+**	1.8	3.3	3.1
SAICAR synthetase [SAICARS (*pur7*)]	Gma.4693.1.S1_at	**-**	**+**	0.1	5.2	5.4
SAICAR lyase [ASAL (*pur8*)]	GmaAffx.86903.1.S1_at	**+**	**+**	3.4	3.6	2.6
AICAR transformylase/IMP synthase [AICART (*pur9/10*)]	GmaAffx.89959.1.S1_at	**+**	**+**	2.8	3.8	4.0
IMP dehydrogenase [IMPDH]	Gma.1685.1.S1_at	**-**	**+**	1.4	6.3	7.1
Adenine phosphorybosyltransferase [APRT]	Gma.5292.1.S1_a_at	**+**	**+**	3.6	4.3	3.5
Adenylate kinase [ADK]	GmaAffx.92905.1.S1_s_at	**+**	**+**	2.8	3.4	3.1
Uricase [Uricase]	GmaAffx.93267.1.S1_s_at	**+**	**+**	3.0	4.1	6.8
Allantoinase [Allantoinase]	GmaAffx.87486.1.S1_at	**-**	**+**	1.2	2.2	1.2

^1 ^+ indicates genes that were detected as differentially-expressed among three different organs after an ANOVA (p < 0.0001, FDR < 0.0015, ≥ 2-fold difference) using the common bean CSH GeneChip data with no masking or after masking with a signal intensity threshold of 80.

^2 ^Log_2 _(nodule/leaf) value obtained from the common bean CSH GeneChip data with no masking or after masking with a signal intensity threshold of 80.

^3 ^ΔΔC_T _= [C_T_(leaf) - C_T_(18s)] - [C_T_(nodule) - C_T_(18s)] from qRT-PCR data. C_T_18s rRNA values were stable in the organs examined and were used to normalize the data.

### Masked probe frequency analysis reveals highly-conserved and highly-divergent gene families between soybean and common bean

The identification of functionally conserved orthologs between species is important because the information obtained for the orthologs in one species, such as soybean, can be readily transferred to the improvement of relative crop species such as common bean. The identification of highly-divergent (rapidly-evolving) genes especially the ones with adaptive divergence between species are also important to better understand the underlying genetic and evolutionary mechanisms. We identified highly-conserved and highly-divergent gene classes between soybean and common bean based on CSH of common bean cRNAs to the soybean GeneChip. Using the masking data obtained in this study we classified the soybean probe sets based on the number of probes retained in each probe set. A total of 1,461 soybean probe sets with 10 or 11 (all) probes retained were classified as a "Highly-conserved" group. A total of 1,551 soybean probe sets with only 1 or 2 probes retained were classified as a "Hyper-variable" group. We did not include the soybean probe sets with all eleven probes masked because those probe sets could be interrogating genes not only with very high sequence variation between the two species but also with low transcript abundance (not expressed).

To validate the classification made based on the number of probes retained per probe set, we examined the sequence homology between soybean probe set target sequences (http://www.affymetrix.com) and the common bean gene index sequences (http://compbio.dfci.harvard.edu/tgi/plant.html) for each class. A total of 1,283 highly-conserved and 1,170 hyper-variable class probe sets had hits after searching against common bean sequences by the BLASTN program with an e-value cutoff of 1e-10. The average number of mismatched nucleotides between two species for the highly-conserved and the hyper-variable group was 18 and 53, respectively. A significantly increased number of nucleotide mismatches for the hyper-variable group compared to the highly-conserved group validates our classification strategy.

**Figure 8:**
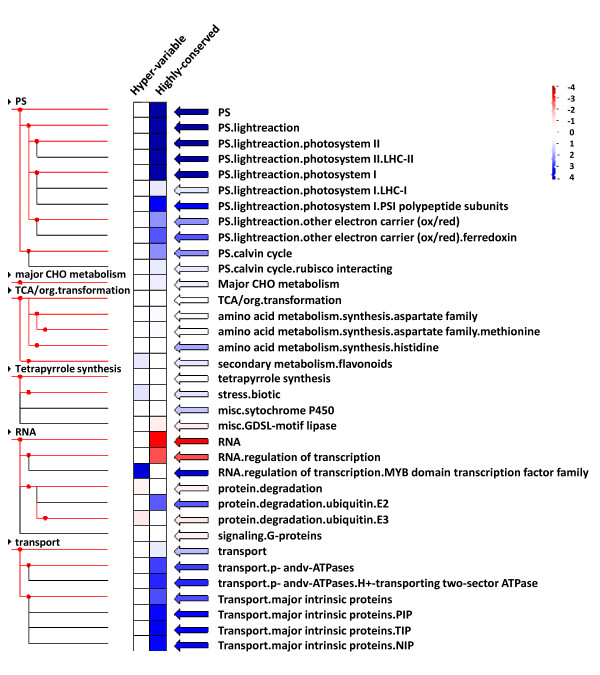
**Over-representation analysis of the hyper-variable and the highly-conserved probe set groups**. The Hyper-variable probe set group retained only 1 or 2 probes, and the highly conserved probe sets group retained 10 or 11 (all) probes after masking with a signal intensity threshold of 80. The Fisher's exact test was performed using the PageMan over-representation analysis module. Over- or under-represented classes in each group were identified after Bonferroni correction (z-value cutoff value = 1.0). The resulting values were then false color coded using a scale of -4 to +4. Blue and red indicate over- and under-representation of the corresponding class, respectively. See methods for details.

We performed a Fisher's exact test with Bonferroni correction (z-value cutoff = 1) to identify functional classes over- or under-represented in highly conserved and hyper-variable groups (see Methods for details). The functional classes over-represented in highly-conserved groups included photosynthesis, protein degradation, and transport classes (Figure 8). Interestingly, the "regulation of the transcription" class (transcription factors) was significantly under-represented among the highly-conserved group (Figure 8). The functional classes over-represented in the hyper-variable group included flavonoid metabolism, biotic stress responsive, and MYB transcription factors (Figure 8). This suggests that the genes for basic cellular functions and metabolisms could be highly conserved between soybean and common bean but there also exists rapidly-evolving classes especially those needed for adaption to the environment. It is widely accepted that orthologous genes in two different species generally (but not necessarily) retain the same function, especially when the function is essential to evolutionary fitness [43]. In addition, the conservation of multiple genes with the same functional class between soybean and common bean observed in this study is consistent with previous reports from other species. Such consistency suggests that conservation at the level of complexes and/or pathways is essential for viability [44-46].

A previous study in *Arabidopsis *showed that rapidly evolving genes under positive selective pressure are more likely to be associated with adaptive divergence between species [47]. In our study, flavonoid metabolism and biotic stress response classes were over-represented among the "hyper-variable" group. These two classes are clearly adaptive in nature. Flavonoids protect plants from microbes and insects [48] and the hyper-variable biotic stress responsive class contains numerous disease resistance (R) genes. Among the hyper-variable biotic stress responsive class of genes, the Toll/interleukin-1 receptors, nucleotide binding sites, and leucine-rich repeat (TIR-NBS-LRR) family of R genes were highly abundant. The NBS-LRR- containing R gene superfamily is one of the largest (~150 genes in *Arabidopsis*) and most diverse gene families in plants [49-51]. The highly divergent (rapidly evolving) and adaptive nature of the R genes can be explained by the high variability in the amino acid sequence of the LRR domain that determines R specificity [49-51].

The MYB transcription factor class was significantly overrepresented among the "hyper-variable" group as well (z-value = 3.6). These transcription factors are involved in various pivotal physiological and developmental processes in plants [52-56]. A previous study that performed a thorough gene expression analysis involving the MYB family reported that most of the *Arabidopsis *MYB genes were responsive to one or multiples types of hormones and stress treatments [56]. The MYB super family is one of the largest gene families in *Arabidopsis *with 198 genes [56,57]. Amplified gene families after multiple gene duplications, like MYB transcription factors, often evolve new biological functions making them highly adaptive in nature.

## Conclusion

The GeneChip^® ^soybean Genome Array is a suitable platform for transcript profiling in common bean. However, data optimization by masking biased probes is necessary to improve sensitivity and accuracy of measuring differential gene expression of the CSH GeneChip data. In addition to transcript profiling, CSH GeneChip data in combination with masking can be used for comparative ecological and/or evolutionary genomics study. This approach could also be an excellent tool for the preliminary examination of diversity patterns between species before high-throughput sequencing. The masking strategy employed in this study can be applied to any species and will assist in answering more fundamental questions such as: 1) phenotypic effects of candidate gene sequence divergence and gene expression patterns, 2) the roles of gene duplication, 3) the relative importance of cis- and trans-acting mutations, and 4) gene expression vs. structural changes for future adaptation.

## Methods

### Plant materials

The common bean (*Phaseolus vulgaris *L.) Mesoamerican cv Negro Jamapa 81 and the Minsoy line of soybean (*Glycine max*) were used in this study. Plants were grown in a growth chamber (27/24°C; 14/10 h photoperiod and 60% of humidity). Surface-sterilized seeds were germinated at 25°C over sterile, wet filter paper. Three day-old seedlings were sown in pots containing Turface MVP (Profile Products LLC, Buffalo Grove, IL). Plants were watered three times per week with Summerfield nutrient solution [58]. After three weeks, leaves and roots were harvested and immediately frozen in liquid nitrogen (N) and stored at -80°C until used for RNA isolation. To evaluate the transcriptional profile of common bean nodules *vs *soybean nodules, three day-old seedlings were sown in pots containing Turface MVP and inoculated with *Rhizobium tropici *CIAT899 for common bean, or *Bradirhizobium japonicum *USDA110. Plants were watered three times per week with N-free Summerfield nutrient solution [58]. After three weeks post-inoculation, nodules were harvested and immediately frozen in liquid N and stored until used for RNA isolation.

### RNA extraction, labeling and GeneChip hybridization

Total RNA was purified from three replicates of leaves, roots or nodules from common bean or soybean plants, using LiCl precipitation protocol as reported in [59].  Contaminating genomic DNA was removed from each sample using the DNA-*free*™ kit following the manufacturer's recommendations http://www.ambion.com. RNA labeling, hybridization, washing and scanning were conducted in the Microarray Facility, Biomedical Genomics Center, University of Minnesota, following standard Affymetrix procedures. Briefly, 10 μg of total RNA was used to synthesize cDNA using SuperScript™ Double-Stranded cDNA Synthesis Kit (Invitrogen, Carlsbad, CA). Biotinylated cRNA was generated via *in vitro *transcription using the Enzo BioArray™ High Yield RNA Transcript Labeling Kit (Enzo Life Sciences, Farmingdale, NY). The Affymetrix GeneChip Sample Cleanup Module was used for cDNA, biotinylated cRNA purification and chemical fragmentation. The integrity of total RNA and fragmented biotin-labeled cRNA was verified with RNA6000 Nano Assay using the Agilent 2100 Bioanalyzer™ (Agilent Technologies, Palo Alto, CA). Labeled cRNA was used for hybridization after 15 μg was fragmented.

### Masking probes targeting variable regions

The source microarray output files used in this study are available in the NCBI Gene Expression Omnibus (http://www.ncbi.nlm.nih.gov/geo/query/acc.cgi?acc=GSE18822) with accession number GSE18822. After scanning the GeneChips, the exported files with probe signal intensities (cel files) were quantile-normalized and background corrected using the affy package from Bioconductor (http://bioconductor.org). Based on the minimums, maximums, and different quantile distributions of the probe signal intensities, we chose a series of signal intensity thresholds (5, 7, 8, 10, 13, 15, 20, 30, 40, 60, 80, 100, 120, 160, 320, 640, 1280, and 2560) for masking to examine which threshold would produce the best performance. One mask file was created for each intensity point. If intensity of the probe was lower than the masking intensity in equal or more than a defined percentage of all cel files, the probe was masked. The defined percentage (P) was determined by the number of replicates in each sample type and the number of sample types:

The *floor *and *ceiling *are mathematical functions that map a real number to the next smallest and next largest integer, respectively. *Ts *is the total number of sample files. R is the replicate number of each sample type. *S *is the number of sample types. In this study, we used leaf, root, and nodule tissue types for each species (*S *= 3) and three replicates (*R *= 3) for a total of 9 sample files (*Ts *= 9), thus *P *= 0.78.

We masked the probes during the RMA probe-level signal summarization process by using Expressionist Refiner module (http://www.genedata.com). Briefly, the raw data cel files were loaded into Refiner with various masking files. The RMA algorithm summarized the probe-level signals into probe set expression indexes. The expression index files produced for the different masking files were further analyzed for performance comparison and gene differential expression analysis using the Expressionist Analyst module.

### Identification of differentially expressed genes

Even though all probes on the soybean GeneChip were used as masking probes with signals below the threshold, the probe sets corresponding to the *Phytophthora sojae *and *Heterodera glycines *were excluded during the identification of differentially expressed genes. Gene expression values were calculated with the RMA [35] as provided with the Genedata Expressionist Pro version 4.5 (Genedata, San Francisco, CA). Genes differentially expressed among different tissues in common bean and soybean were identified by ANOVA (*p*-value < 0.0001, FDR < 0.0015) with an additional cutoff of a 2-fold ratio in pair-wise comparisons (i.e., nodule vs. leaf; nodule vs. root; root vs. leaf).

### Functional classification and over-representation analysis

MapMan software was originally developed to visualize *Arabidopsis *microarray data in multiple metabolic pathways and provide an overview of cellular function and regulation [39]. A previous study developed MapMan bins for the soybean GeneChip based on the Kyoto Encyclopedia of Genes and Genomes (KEGG) database [60]. Since the soybean MapMan bin system previously introduced was not compatible with the original bins developed for *Arabidopsis*, the original figures provided with the MapMan software could not be used. To fully utilize resources provided by the MapMan software, we developed a customized MapMan bin for the soybean GeneChip. To adapt MapMan to the soybean GeneChip system, soybean GeneChip probe set consensus sequences (http://www.affymetrix.com/index.affx) were compared with the *Arabidopsis *proteome using BLASTX with an E-value cutoff of 10^-10^. The 34 major MapMan BINs and their subBINS for the top *Arabidopsis *hits were assigned to each probe set in the soybean GeneChip. Putative transcription factor families on the soybean GeneChip were further identified using a BLASTX search (E-value < 10^-10^) against 1827 protein sequences consisting of 56 transcription factor families from the *Arabidopsis *transcription factor database (http://datf.cbi.pku.edu.cn/index.php). After the first round of classification, additional MapMan bins were manually assigned, if needed, via text search for GO, KEGG, COG, and MIPS ontology annotation for each probe set utilizing the information available at the GeneBins database [60]. Additionally, probe sets for putative nodulin (or nodulin-like) genes were identified using known nodulin protein sequences (E-value < 10^-10^) in the public database. The new subBIN 26.31, misc.nodulins, was assigned following Tellström et al. [61]. Otherwise the sequence was classified as "not assigned".

PageMan, a software tool for comparative analysis of gene ontology, was originally developed to display and annotate overview graphs for profiling experiments [62]. We adapted PageMan to perform functional class over-representation analysis of selected genes compared to the whole soybean probe sets in the soybean GeneChip. For highly conserved and hyper-variable genes, the selected probe sets were given a value of "1", for differentially expressed genes, the selected probe sets were given their log ratio values [i.e. log_2_(nodule/leaf)], and the rest of the probe sets were given a value of "0" as a false expression value. The data were then loaded into PageMan. The Fisher's exact test with Bonferroni correction with a z-value cutoff value of "1" was applied to detect functional classes over- or under-represented among genes selected. The adjusted *p*-values produced were then transformed into their respective z-values. The resulting values were then false color coded using a color scale of -4 to 4. Blue and red indicate over- and under-representation of the corresponding class, respectively.

### Real-time quantitative RT-PCR

A portion of the total RNA used for the GeneChip hybridization step was used to make cDNAs for qRT-PCR. Contaminating genomic DNA was removed from each sample using the DNA-*free*™ kit following the manufacturer's recommendations (http://www.ambion.com). The first strand cDNA for each sample was made using random hexamers and Taqman Reverse Transcription Reagents (Applied Biosystems, CA) following the manufacturer's recommendations. Gene specific primers were subsequently designed using Primer Express (Applied Biosystems, CA) (Additional file 4). The specific primer sequences were based on the probe set consensus sequences (http://www.affymetrix.com) or sequences from the common bean gene index (http://compbio.dfci.harvard.edu/tgi/plant.html), if the corresponding bean gene ortholog sequence was available. Samples and standards were run in triplicate on each plate and repeated on at least two plates using SYBR-Green PCR Master Mix (Applied Biosystems, CA) on a GeneAmp 7000 Sequence Detection System (Applied Biosystems, CA) following the manufacturer's recommendations. qRT-PCR was performed in a 25 μl reaction containing 6.5 μl ddH2O, 12.5 μl 2× PCR mix, 1 μl forward primer (1 μM), 1 μl reverse primer (1 μM), and 4 μl of template cDNA (5 ng/μl). The PCR conditions were as follows: two minutes of pre-incubation at 50°C, 10 minute of pre-denaturation at 94°C, 40 cycles of 15 seconds at 95°C and one min at 60°C, followed by steps for dissociation curve generation (30 seconds at 95°C, 60 seconds at 60°C and 30 seconds at 95°C). The GeneAmp 7000 System SDS software v.1.2.2. was used for data collection and analysis. Dissociation curves for each amplicon were carefully examined to confirm lack of multiple amplicons at different melting temperatures (Tms). Relative transcript levels for each sample were obtained using the "comparative C_T _method" [63]. The threshold cycle (C_T_) value obtained after each reaction was normalized to the C_T _value of the 18S rRNA. The relative expression level was obtained by calibrating the ΔΔC_T _values for common bean leaf samples using a normalized C_T _value (ΔΔC_T_) for the nodule common bean sample.

## Abbreviations

CSH: cross-species hybridization; EST: expressed sequence tag; MAS: microarray suite; PCA: principle component analysis; RMA: robust multi-array average; ISV: inter-species variable; SNP: single nucleotide polymorphism.

## Authors' contributions

SY and WX performed the computational analysis involved in the electronic masking of probes. SY conducted functional classification, over-representative analysis and qRT-PCR validation. OV and BB performed the microarray work for the generation of GeneChip data. CV and GH designed the experiment. All authors contributed to the analysis of results and writing of the manuscript.

## Supplementary Material

Additional file 1 **Soybean genes differentially expressed among nodule, root, and leaf**. A table listing genes (probe sets) that were differentially expressed among nodule, root, and leaf (p-value < 0.0001, FDR < 0.0015, ≥ 2 fold difference) including corresponding p-values, FDR values, log ratios, and MapMan functional classes for soybean. Click here for file

Additional file 2 **Common bean genes differentially expressed among nodule, root, and leaf**. A table listing genes (probe sets) that were differentially expressed among nodule, root, and leaf (p-value < 0.0001, FDR < 0.0015, ≥ 2 -fold difference) including corresponding p-values, FDR values, log ratios, and MapMan functional classes for common bean. Click here for file

Additional file 3 **Functional classes over-representation analysis for genes up-regulated in nodule tissue compared to leaf and root tissue in soybean and common bean**. A figure showing the gene functional class over-representation analysis result for genes up-regulated in nodule tissue compared to leaf and root tissue in soybean and common bean. For details see the Fig. 8 legend. Click here for file

Additional file 4 **Primers used for qRT-PCR validation**. A table listing primers used for qRT-PCR validation of 20 randomly selected genes and the 13 purine-ureide pathway genes. Click here for file
